# Uncomfortable images produce non-sparse responses in a model of primary visual cortex

**DOI:** 10.1098/rsos.140535

**Published:** 2015-02-25

**Authors:** Paul B. Hibbard, Louise O'Hare

**Affiliations:** 1 Department of Psychology, University of Essex, Wivenhoe Park, Colchester CO4 3SQ, UK; 2 School of Psychology and Neuroscience, University of St Andrews, St Andrews, Fife KY16 9JP, UK; 3 School of Psychology, University of Lincoln, Lincoln, UK

**Keywords:** visual discomfort, natural images, sparse coding, kurtosis

## Abstract

The processing of visual information by the nervous system requires significant metabolic resources. To minimize the energy needed, our visual system appears to be optimized to encode typical natural images as efficiently as possible. One consequence of this is that some atypical images will produce inefficient, non-optimal responses. Here, we show that images that are reported to be uncomfortable to view, and that can trigger migraine attacks and epileptic seizures, produce relatively non-sparse responses in a model of the primary visual cortex. In comparison with the responses to typical inputs, responses to aversive images were larger and less sparse. We propose that this difference in the neural population response may be one cause of visual discomfort in the general population, and can produce more extreme responses in clinical populations such as migraine and epilepsy sufferers.

## 2. Introduction

The high metabolic cost of neural computation means that it is only possible for a small fraction of cortical neurons to be active at any one time. Lennie [1] estimated that, in the visual cortex, this fraction is less than 2%. It is therefore important that visual information is encoded efficiently. One way of accomplishing this is to ensure a sparse distribution of responses across the population of cortical neurons. This is a response in which information is conveyed by strong activity in a small proportion of neurons, while the majority remain relatively inactive. It is possible to create metabolically efficient, sparse responses to natural images [2] by exploiting their statistical redundancy [3]. There is clear evidence that the mammalian visual system responds sparsely to natural images. For example, models of populations of neurons with properties similar to those found in the visual cortex have been shown to produce sparse responses to natural image inputs [4]. Equally, learning algorithms that seek to generate sparse responses to natural image samples produce units with receptive fields that are strikingly similar to those found in the visual cortex [5]. This suggests that sparse coding might, indeed, be a strategy used by the human visual system to maximize information transfer with minimum metabolic cost. It is important to note, however, that an encoding that produces sparse responses to natural images may respond non-sparsely to other inputs.

Most research on natural image statistics has sought to establish how efficient coding of this type is achieved. Such research has proved extremely valuable in understanding visual processing across many dimensions, including luminance, contrast, colour, motion [6] and binocular disparity [7]. One property typical of natural images is that they show little variation in their spatial frequency content: typically, this can be characterized as a 1/*f*^*β*^ amplitude spectrum, where *β* varies between 0.8 and 1.2 [8]. Natural images with these properties are generally judged to be comfortable to look at [9]. There is also a growing literature suggesting that artwork is pleasing to the eye as its statistical properties occupy a narrower range within this bracket, and therefore these stimuli can be even more optimally, and sparsely, encoded [9–13]. An important, and hitherto neglected consequence of this specialization is that the responses to images with atypical statistical properties will be non-optimal. Thus, some images will exist that create inefficient, non-sparse responses. Here, we show that images that create discomfort, and that can trigger epileptic seizures [14] and migraine attacks [15], produce relatively non-sparse responses.

**Figure 1.**
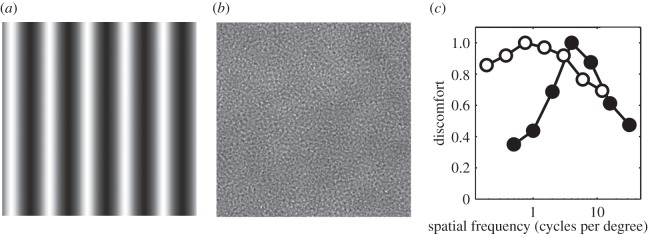
(*a*) A sine grating. (*b*) A filtered noise pattern. (*c*) Results of discomfort studies replotted: filled circles represent discomfort judgements of striped patterns of varying spatial frequency [17], open circles represent discomfort judgements of filtered noise patterns of varying spatial frequency content [18]. The results have been normalized to a maximum value of 1.

It is well established that images with some types of statistical structure produce adverse reactions, including headaches, eye-strain and illusions of shape, colour and motion, when viewed, which is referred to as ‘visual discomfort’ [16]. Such images have excessively high amplitude at midrange spatial frequencies in comparison with natural images. In particular, striped gratings (see figure 1*a* for an example) with a spatial frequency within an octave of four cycles per degree have been found to be more uncomfortable than ones with a higher or lower spatial frequency [17]. These findings are replotted in figure 1*c*. Spatial frequency content is also critical to the amplitude of the cortical response in epilepsy sufferers [19]. The spatial frequency content of images other than striped patterns is important in determining the degree of visual discomfort that they will induce [20,21]. Filtered noise patterns (see figure 1*b* for an example) with spatial frequency content typical of natural images tend to be judged as more comfortable than those with spatial frequency content that deviates from that typical of natural images [18,20,21], as shown in figure 1*c*.

Here, we show that the types of images that create visual discomfort, and that trigger migraine attacks and epileptic seizures, will produce excessively large, non-sparse responses in the primary visual cortex. To do this, we apply and modify the techniques pioneered by Field [4] in the analysis of the visual system's responses to natural images. Field argued that the goal of sensory coding is to produce a sparse response, across the population of cortical visual neurons, to natural images. Field modelled cortical neurons as a population of log-Gabor wavelet functions. These functions, which have receptive fields that are localized in space and tuned to spatial frequency and orientation, provide an accurate model of simple cells in cortical area V1. Field [4] calculated the responses of populations of these model neurons to the raw information present in natural images and to a model population of retinal neurons. Field found that subsequent stages of neural processing created increasingly sparse responses to natural images. We used this approach to assess the magnitude and sparesness of responses to the types of artificial images that have been associated with visual discomfort. Our logic is that, if it is important that the population response of visual neurons to natural images is sparse, then those stimuli that create non-sparse responses will be challenging for the visual system to encode. In particular, stimuli that create large, non-sparse responses would place excessive metabolic demands on the visual system. It is these excessive demands that are associated with discomfort [22], and are increased in migraine [23,24].

In our analysis, we made an important change to the modelling approach developed by Field [4]. In his analysis, images were convolved with 16 filters, with tuning to two spatial frequencies, four orientations and two phases. This can be used to simulate the population response of neurons with this range of tunings, at all locations in the image. It is important to remember that the responses of individual filters to each image are jointly determined by the image and the tuning of the filter. The population response therefore depends crucially on the tunings of the filters. While the log-Gabor filters used by Field [4] were carefully chosen to match the shapes of the receptive fields of cortical neurons, the distribution of the tunings of the filters to parameters such as orientation and spatial frequency was not. This is an important consideration, because the distributions of these parameters will have a strong influence on both the magnitude and population sparseness of the neural response.

**Figure 2.**
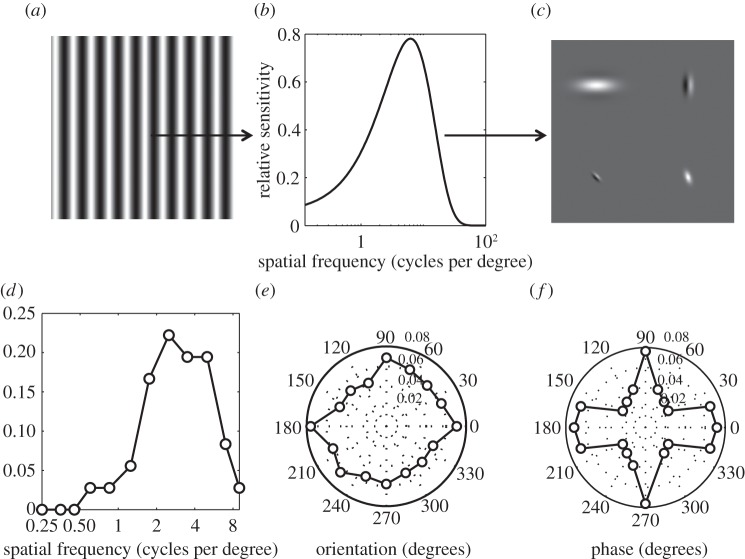
The stimulus (*a*) was filtered to take account of the contrast sensitivity function (*b*), before filtering with (*c*) model neurons with Gabor receptive fields. The probability distributions of tuning for (*d*) spatial frequency (*e*) orientation and (*f*) phase were taken from physiological data.

We modelled the responses of populations of neurons with distributions of frequency, orientation and phase tunings based on those found in physiological recordings. These are shown in figure 2*d*–*f*. Of particular interest in the current context is that the distribution of spatial frequency tunings is very non-uniform, with many more neurons tuned to midrange frequencies around two to six cycles per degree. This means that images in which power is concentrated around these frequencies will produce strong responses in many neurons, resulting in large, non-sparse population responses. Our analysis provides a potential explanation for why images with these characteristics are associated with both large haemodynamic and electromagnetic responses, and visual discomfort.

## 3. Methods

### 3.1 Stimuli

#### 3.1.1 Sinewave gratings

Pixel images of sinewave gratings (1024×1024) were created, with a resolution of one pixel per arc min of visual angle. Gratings with spatial frequencies of 0.25, 0.5, 1, 2, 4, 8 and 12 cycles per degree were used.

#### 3.1.2 Filtered noise

Filtered noise stimuli were created following previous work on visual discomfort [20,18]. Pixel Gaussian white noise images (1024×1024) were created, again with a resolution of 1 pixel per arc min. These images were then filtered in the Fourier frequency domain to have a 1/*f* amplitude spectrum, with a peak at a particular frequency that was created by multiplication with a raised radial cosine filter:

3.1 $$\begin{array}{rl} H(f) & =\left\{ \begin{array}{l} T\\ \frac{T}{2}\left[1+\cos\left(\frac{\pi T}{\beta }\right)\left(|\log(f)-\log(f_{0})|-\frac{1-\beta }{2T}\right)\right]\\ O \end{array}\right.\\ & \;\text{for}\;\left\{ \begin{array}{l} \left(0\leq |\log(f)-\log(f_{0})|\leq \frac{1-\beta }{2T}\right)\\ \left(\frac{1-\beta }{2T}\leq |\log(f)-\log(f_{0})|\leq \frac{1+\beta }{2T}\right)\\ \left(|\log(f)-\log(f_{0})|>\frac{1+\beta }{2T}\right), \end{array}\right. \end{array}$$

where *T* is 0.9, *β* is the roll-off factor of 0.5, *f* is spatial frequency and *f*_0_ is the centre frequency of the peak, ranging from 0.25 to 12 cycles per degree.

#### 3.1.3 Natural images

One hundred natural images were sampled at random from the van Hateren database [5]. Images with a linear luminance function were used.

### 3.2 Model neurons

V1 cortical cells were modelled using Gabor functions:

3.2 $$G(k)=A\exp\left(-\frac{x_{p}^{2}}{2\sigma_{1}^{2}}-\frac{y_{p}^{2}}{2\sigma_{2}^{2}}\right)\cos(2\pi fx_{p}+\phi ),$$

where *σ*_1_=7.81/*f* and *σ*_2_=15.61/*f*, and *y*_*p*_ and *x*_*p*_ are defined as

3.3 $$\begin{array}{rl} x_{p} & =x\cos\theta -y\sin\theta \end{array}$$

3.4 $$\begin{array}{rl} y_{p} & =x\sin\theta +y\cos\theta . \end{array}$$

Here, *f* is the preferred spatial frequency of the model cell, *θ* the preferred orientation and *ϕ* is the phase. For each stimulus, 250 000 model neurons were created.

For natural images, the spatial frequency, orientation and phase tunings of the neurons were set in two ways. In the first simulation, we used the values for these parameters that were used by Field [4]. We used filters tuned to two spatial frequencies (20 and 40 cycles per image), four orientations (10, 55, 100 and 145 degrees) and two phases (0 and 90 degrees).

In our second simulation, the distributions of the tuning of our model neurons for spatial frequency [25], orientation [26] and phase [27] were based on physiological data from single cell recordings (figure 2*d*–*f*). The data for spatial frequency were recorded from neurons with receptive fields in the fovea in macaque monkeys. The data for orientation were recorded from neurons with receptive fields in the central 15 degrees of the visual field in cats. The data for phase were recorded in the macaque; the locations of the receptive fields were not reported in this study. The values of *σ*_1_ and *σ*_2_ used produced a spatial frequency bandwidth of 1.4 octaves, and receptive fields that were two times longer in the direction parallel to the orientation tuning of the filter than in the orthogonal direction. For each stimulus, we calculated the distribution of the responses of our model neurons. We were interested in both the magnitude and sparseness of the population response. Magnitude was simply the total response across all model neurons, and sparseness was quantified as the kurtosis of the population response [4]. For distributions such as ours, which are peaked around zero, high kurtosis represents a sparse population response, in which the majority of neurons respond weakly. Conversely, a low kurtosis indicates a less sparse response.

### 3.3 Simulating neural responses

To calculate the responses of the model neurons, the receptive field was positioned at a randomly chosen location in the image. The luminance value for each pixel within the receptive field was multiplied by the receptive field's weighting function at that point, and all such values were summed across space to give the neuron's response.

In the second simulation, in which we compared responses with sinewave and filtered-noise stimuli with responses to natural images, we pre-filtered our images to take account of the contrast sensitivity function [28]. This is an important characteristic of the early visual system, and is determined, in part, by the optical modulation transfer function, and the bandpass filtering that occurs in the retina [29,30]. These pre-cortical filtering stages will affect the population response in ways that depend on the spatial frequency content of the stimuli, and it is important therefore to understand the contribution of this filtering, as well as the distribution of cortical tuning for spatial frequency, on the results that we obtained. We therefore performed a third simulation in which we did not attempt to take the contrast sensitivity function into account. All images were first scaled, so that the luminance values varied between 1 and 255. The original Field model was applied just to natural images, to allow for a comparison between the two models with different tunings. The modified model (second and third simulation) was applied to all of the stimuli. All simulations were performed using MATLAB.

## 4. Results

**Figure 3.**
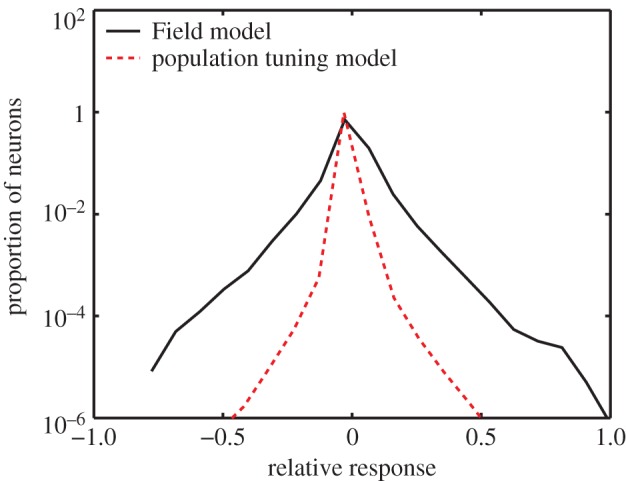
(*a*) The distribution of response magnitudes, across the population of model cells, to natural images. The black line shows the responses for the model with the tuning to spatial frequency, orientation and phase of the original [3] model. The red dotted line shows the responses for the revised model, with these tunings based on physiological data.

Figure 3 shows the distribution of responses to natural images, simulated using the original Field model parameters, and also the modified model, which has parameters based on physiological data. For both models, there is an overall peak in the response at zero, showing that the activity of most units is low. The responses of our modified, physiology-based model are clearly more concentrated around zero than those for the original, unmodified model. This is reflected in both the mean of the absolute values of the responses (53% of the value of those for the original Field model parameters) and the population excess kurtosis (149 for the modified model, 6.2 for the original model). These results suggest that the sparesness of neural population responses, when the distributions of cells are taken into account, is much greater than suggested by Field's original model. These results demonstrate the important role played by the combination of the statistics of the input image and the properties of cortical filters in determining the nature of the neural response.

**Figure 4.**
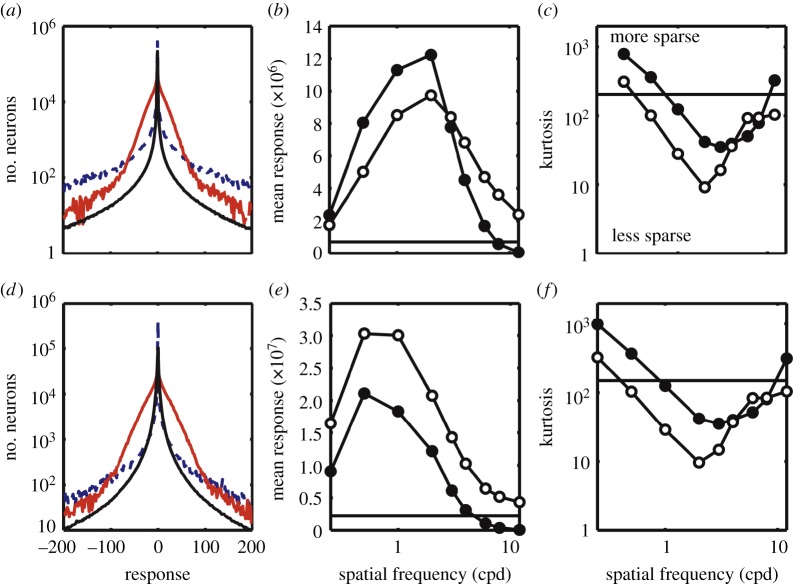
(*a*) The distribution of response magnitudes, across the population of model cells. The solid black line shows responses to natural images, the solid red line responses to sine gratings and the dashed blue line responses to filtered noise stimuli. In the latter two cases, results are shown for a three cycles per degree stimulus. For all stimuli, the response is strongly peaked at zero. Both sine grating and filtered noise stimuli show broader distributions, indicating that a greater number of cells are producing strong responses. (*b*) The total magnitude and (*c*) the kurtosis of the population response. Results are plotted separately for sinewave (filled circles) and filtered noise (open circles) stimuli. The solid horizontal line shows the mean responses to 100 natural images. For both stimuli, clear tuning is observed in each metric. Panels (*d*), (*e*) and (*f*) show the same results for images that were not filtered to take account of the contrast sensitivity function.

The overall distribution of modified model responses to natural images, gratings and filtered noise can be seen in figure 4*a*. Figure 4 shows results only for the model in which the tuning functions of neurons are based on physiological data. For all classes of stimuli, there is an overall peak in the response at 0, showing that the activity of most units is low. The shapes of the distributions are broadly similar. This similarity to the responses to natural images is critical in allowing us to compare measures of response kurtosis and overall response magnitude. The response magnitude for both grating and filtered noise stimuli was generally larger than for natural images, and depended on spatial frequency, as shown in figure 4*b*. The magnitude peaks at two cycles per degree for both stimuli. This is in close agreement with discomfort judgements [17,18]. The response kurtosis shows that this increase in response magnitude partly reflects a response that is less sparse. The kurtosis of the population response is shown in figure 4*c*. Kurtosis showed clear spatial frequency tuning, with the least sparse response occurring at three cycles per degree for sinewaves, and at two cycles per degree for filtered noise. Thus, uncomfortable images tended to produce excessive, non-sparse responses in comparison with typical natural images.

In the second simulation, we pre-filtered our images to take account of the contrast sensitivity function. To determine the importance of this filtering, the simulations were repeated without it. This third simulation produced similar results (figure 4*d*–*f*), indicating that our findings reflect the interaction between the properties of the input stimuli and the model architecture. For both types of stimulus, the tuning of response magnitude now peaked at a lower spatial frequency, reflecting the importance of the contrast sensitivity function. Our results thus reflect the combined effects of contrast sensitivity, and the distribution of spatial-frequency tuning of cortical neurons.

## 5. Discussion

All of the model response distributions showed a similar shape to that obtained with natural images [4], however response magnitude and kurtosis differed, depending on the input stimuli. The population response kurtosis was used as a measure of the population sparesness [4]. The total magnitude of the population response was also calculated, as a large cortical response has been proposed as a possible cause of discomfort [21]. These are measures of the distribution and magnitude of responses across the population of neurons, to a particular input stimulus. This is in contrast to measures of the lifetime sparseness and response magnitude, which can be used to characterize the responses of each individual neuron to different input stimuli. Because we are interested in characterizing the overall cortical response, and how this differs across images, it is measures of the population response that are of interest in the current context.

Our results indicate that stimuli previously judged to be uncomfortable produce both higher responses and reduced sparesness compared with natural images. This provides a potential explanation for visual discomfort in both normal and clinical populations. Excessive responses to uncomfortable images have been found using both fMRI [23] and MEG [22]. In clinical cases, these excessive responses may be the cause of both migraine attacks and epileptic seizures.

The model was an extension of the work of Field [4], to include the relative distributions of cortical cells, and a gain control weighting function to represent the contrast sensitivity function. The location of the peak of the response distribution was affected by the contrast sensitivity function, but not the overall shape of the response. This could go some way to explaining the variation in discomfort judgements—some studies find the maximum discomfort around four cycles per degree [17], others slightly lower [24]. This could potentially be explained by differences in contrast in the stimuli.

A sparse code represents one possible method of efficient coding. One limitation of this model is that it does not address the decoding part of the information transfer, which is important for the system to recognize what is in the image, for example. In order to be useful, information that is sufficient to accurately undertake subsequent tasks must be transmitted. This is a much more difficult problem, which has not yet been answered. Additionally, there are no data on how much information the visual system can transmit with a given amount of metabolic energy ([31, pp. 77–78]). Lennie [1] suggested 1/50th of the units can be strongly active, but this tells us nothing of the quality of information transmitted.

In summary, as natural images are optimally processed by the visual system, we demonstrate that, in a model of primary visual cortex, stimuli that have previously been shown to produce discomfort produce large, non-sparse responses. It has been suggested that visual discomfort may be the result of multiple causes [16]. We propose that one such cause is stimuli that are challenging and metabolically costly to process.

## Data accessibility

MATLAB code is available from figshare: http://dx.doi.org/10.6084/m9.figshare.1288820.
